# Investigating Mechanisms of Alkalinization for Reducing Primary Breast Tumor Invasion

**DOI:** 10.1155/2013/485196

**Published:** 2013-07-10

**Authors:** Ian F. Robey, Lance A. Nesbit

**Affiliations:** Arizona Respiratory Center, University of Arizona, 1501 N. Campbell Avenue, Suite 2349, P.O. Box 245030, Tucson, AZ 85724, USA

## Abstract

The extracellular pH (pHe) of many solid tumors is acidic as a result of glycolytic metabolism and poor perfusion. Acidity promotes invasion and enhances metastatic potential. Tumor acidity can be buffered by systemic administration of an alkaline agent such as sodium bicarbonate. Tumor-bearing mice maintained on sodium bicarbonate drinking water exhibit fewer metastases and survive longer than untreated controls. We predict this effect is due to inhibition of tumor invasion. Reducing tumor invasion should result in fewer circulating tumor cells (CTCs). We report that bicarbonate-treated MDA-MB-231 tumor-bearing mice exhibited significantly lower numbers of CTCs than untreated mice (*P* < 0.01). Tumor pHe buffering may reduce optimal conditions for enzymes involved in tumor invasion such as cathepsins and matrix metalloproteases (MMPs). To address this, we tested the effect of transient alkalinization on cathepsin and MMP activity using enzyme activatable fluorescence agents in mice bearing MDA-MB-231 mammary xenografts. Transient alkalinization significantly reduced the fluorescent signal of protease-specific activatable agents *in vivo* (*P* ≤ 0.003). Alkalinization, however, did not affect expression of carbonic anhydrase IX (CAIX). The findings suggest a possible mechanism in a live model system for breast cancer where systemic alkalinization slows the rate of invasion.

## 1. Introduction

The extracellular microenvironment of most solid tumors is acidic [1]. Extracellular pH (pHe) is typically between 6.5 and 6.9. Normal tissue pHe resides between 7.2 and 7.5 [1–3]. Tumor acidity may help drive malignancy because it is known to directly stimulate activity of matrix remodeling enzymes such as metalloproteases (MMP1, MMP-2, and MMP-9) [4–6] and lysosomal proteases such as cathepsin B, D, or L [7–9]. Furthermore, acidification of culture media can produce more invasive tumor cells *in vitro* and produce metastases more readily *in vivo* [4, 5, 10, 11]. These observations support the “acid-mediated invasion hypothesis” which posits tumor acidity as a driving microenvironmental component of tumor cell invasion and extracellular matrix (ECM) degradation of the parenchyma encapsulating primary tumors [12–14].

Previous studies have shown that buffering tumor acidity through systemic administration of an alkaline agent raises the pHe of tumors [15, 16]. The ability to modulate tumor pHe has led to studies demonstrating the biological impact of alkalinization on cancer progression and survival in mice. The findings agree, at least in some tumor types, that systemic alkalinization reduces the spread of metastases and improves survival in tumor-bearing animals [16–19]. The mechanisms driving these observations, however, are not well understood. 

We hypothesize that alkalinization of the primary tumor has the effect of reducing invasion. To explore this question, we investigated the effect of chronic alkalinization on intravasation by evaluating the frequency of circulating tumor cells (CTCs) in mice bearing MDA-MB-231 mammary xenografts. The study also examined the potential for alkalinization to inhibit the invasion processes of tumor cells by reducing the activity of pH-sensitive proteases involved in ECM remodeling. We tested the relationship between alkalinization and protease activity by measuring the fluorescence signal from protease activatable agents pertaining to cathepsins and MMPs in response to transient alkalinization in tumor-bearing mice. Imaging experiments also tested the effect of alkalinization on CAIX tumor expression. 

## 2. Methods & Materials 

### 2.1. Mouse Experiments

Mice were handled in accordance with Institutional Animal Care and Use Committee-approved protocols at the University of Arizona. Four eight-week-old female SCID (Arizona Cancer Center breeding colony; C.B-17/IcrACCscid) or nude mice (Taconic Farms; CrTac:NCr-Foxn1) received orthotopic injections of 5 × 10^6^ MDA-MB-231 tumor cells in the mammary fat pad. The MDA-MB-231 cells were stably transfected to express neomycin-resistant pcDNA3/EGFP plasmid. These cells constitutively express green fluorescent protein (GFP) which exhibits emission fluorescence in the 520 nm range. As stated in a previous publication, these cells were a gift from Peter Ratcliffe, Oxford University, Oxford, UK [16]. For CTC experiments, tumor bearing mice were randomized into an untreated or treated cohort one day after tumor cell inoculation. The treated cohort received 200 mM sodium bicarbonate (Sigma, St. Louis, MO, USA) drinking water which is equivalent to 3.2 g/kg/day in mice. Mice were monitored and maintained by the Experimental Mouse Shared Services core facility of the University of Arizona, Tucson, Arizona. Volumes of primary tumors in mammary fat pads were measured twice weekly and calculated from orthogonal measurements of external dimensions as (width)^2^  ×  (length)/2.

Blood for CTC counting was collected by heart puncture from tumor bearing SCID mice when tumor burden reached ~1,800 mm^3^ at about 9 weeks after xenografts were established. Animals were euthanized by cervical dislocation. In separate experiments, fluorescence imaging was carried out when tumors were palpable at 100 mm^3^. For urine pH experiments, SCID mice received 21 mg or 84 mg of sodium bicarbonate p.o. Urine was obtained by applying gentle abdominal pressure against the mouse for 10 sec over plastic film. Urine was collected by micropipette and transferred to a 0.6 mL tube for pH measurement. Urine pH was measured using a pH meter with an InLab Micro Probe (Mettler Toledo, Columbus OH). 

### 2.2. Flow Cytometry

Prior to blood collection procedure, mice were subjected to CO_2_ asphyxiation. Blood (~0.8 mL) was collected by cardiac puncture of the right ventricle using a 22-gauge needle and 1 mL syringes. The needle dead space was filled with EDTA (100 mmol/L) to prevent coagulation. Red blood cells were lysed by incubating whole blood preparations in 1 mL of BD FACS lysing solution (Becton Dickinson, Franklin Lakes, NJ, USA) for 15 minutes at room temperature, while gently inverting the mixtures. Lysed blood cells were removed from supernatant of samples after 5 minute 200 ×g centrifugation. The pellets were resuspended in 100 *μ*L of phosphate buffered saline (PBS; Cellgro, Manassas, VA, USA) and incubated with anti-mouse phycoerythrin-labeled (PE) CD45 (3 *μ*L) (eBioscience, San Diego, CA, USA) for 15 min at room temperature away from light. Cells were washed in PBS and incubated 30 minutes on ice and away from light with 1 *μ*L Fixable Viability Dye eFluor 660 (eBioscience). The samples were washed and centrifuged in PBS, and then suspensions were fixed with IntraPrep fixation reagent (Beckman Coulter, Fullerton, CA, USA) according to the manufacturer's instructions. After washing, samples were resuspended in 0.5 mL of PBS and kept on ice until flow cytometry procedure. 

Flow cytometry was carried out using a FACSCanto (Becton Dickinson) flow cytometer. Flow settings for the experiments were precalibrated using sets of whole blood from non-tumor bearing mice treated with 10,000, 1000, 100, 10, or 0 MDA-MB-231-GFP cells. Cells of interest were defined first using forward and side scatter parameters. Large MDA-MB-231-GFP cells were detected using the FL1 channel (525 nm bandpass filter). Cells exhibiting a PE-positive signal in the FL2 (575 nm bandpass filter) channel were negatively gated as CD45-expressing mouse leukocytes. Criteria-positive events were collected at 50,000 cells per sample. CTC events were expressed as counts per mL of blood. Flow cytometry analysis was carried out using the FACSDiva software program (Becton Dickinson).

### 2.3. *In Vivo* Fluorescence Imaging

The fluorescent (near-infrared) probes used in these experiments were MMPSense 750 FAST that detects matrix metalloprotease activity, ProSense 750 FAST that detects pan-cathepsin (B, L, S, K, V, and D) activity, and HypoxiSense 680 that targets carbonic anhydrase IX (CAIX) (PerkinElmer, Boston, MA, USA). Mice received a 100 *μ*L tail vein injection of 2–4 nmol fluorescent agent 24 hours prior to imaging experiments. Mice (*n* = 6) were anesthetized using 3%, O_2_ 2 L/min isoflurane in O_2_ carrier gas at 2 L/min and then placed in a light-free imaging box (Spectral Instruments Imaging, Tucson, AZ, USA) with nose cones supplying 1.5% isoflurane to anesthetized mice during imaging. Image acquisitions consisted of a 5 sec exposure for near-infrared probes (745/810 nm excitation/emission wavelength) and 1 sec exposure for GFP (465/530 nm). Fluorescence images were overlaid on light images of the mice. After time 0 (pretreatment) images, mice were administered either PBS or sodium bicarbonate (42 mg or 84 mg). Administration of agents was delivered by oral gavage (p.o.) (0.25 mL) or through intraperitoneal (i.p.) injection (1 mL). The collected fluorescence images from sequential time points in the individual experiments were adjusted to have identical minimums, maximums, and threshold values. A freehand ROI was drawn around the visible fluorescence regions. The photon counts in the ROIs were defined in this study as relative fluorescence units (RFU). RFU was calculated by normalizing the photon counts from each time point to the pretreatment time point (100%) in each mouse. Due to variability between fluorescence values in each mouse at time 0, the ROI values at different time points were normalized only to the individual mouse and not to a mean pretreatment value. The ROI analyses were carried out using the ImageJ program (http://rsb.info.nih.gov/ij/) to verify the results.

### 2.4. Statistics

Statistical calculations were conducted using the analysis feature in GraphPad Prism version 4.03 for Windows (GraphPad Software, San Diego CA, USA, http://www.graphpad.com). Unpaired, two-tailed *t*-tests were used to determine if means were significantly different between untreated and treated groups. A *P* value of less than 0.05 was considered to be statistically significant. All data values were presented as mean ± SEM.

## 3. Results

### 3.1. Sodium Bicarbonate-Treated Mice Exhibit Lower Circulating Tumor Cells (CTCs)

**Figure 1:**
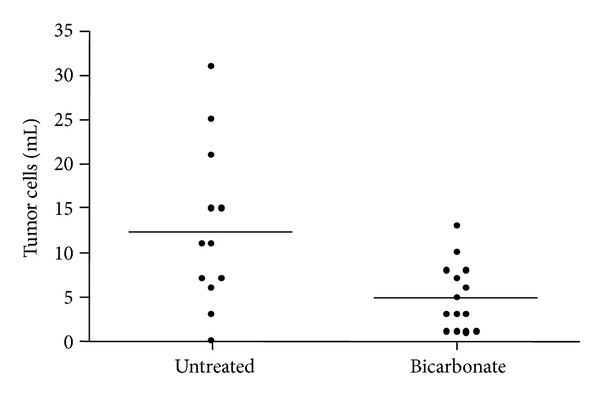
Circulating tumor cell frequency in sodium bicarbonate-reated and untreated mice. Tumor bearing mice were maintained on oral sodium bicarbonate (200 mM) or water at time of tumor inoculation. Tumors were allowed to grow to an average of 1,800 mm^3^ in volume (approximately 9 weeks) before testing blood for CTCs. MDA-MB-231-GFP tumor cells were counted by flow cytometry analysis. There were significantly fewer CTCs in bicarbonate-treated mice compared to untreated mice (*P < 0.01).

To determine if systemic alkalinization affects CTC frequency, SCID mice bearing MDA-MB-231-GFP xenografts were randomized into a treatment group that was administered 200 mM sodium bicarbonate drinking water (*n* = 14) and an untreated (*n* = 12) group. This regimen was maintained for 9 weeks until primary tumors grew to approximately 1,800 mm^3^. Large, GFP expressing cells were positively sorted from blood preparations and enriched by negatively gating on mouse leukocyte marker CD45. The mean CTC count in untreated and bicarbonate-treated mice was 13 ± 3 and 5 ± 1 cells/mL blood, respectively (Figure 1). The frequency of CTC in blood of mice treated with sodium bicarbonate drinking water was significantly less than half of that of untreated mice (*P* < 0.01).

### 3.2. Bolus Dosing with Sodium Bicarbonate Produces Transient Urine pH Alkalinization

**Figure 2:**
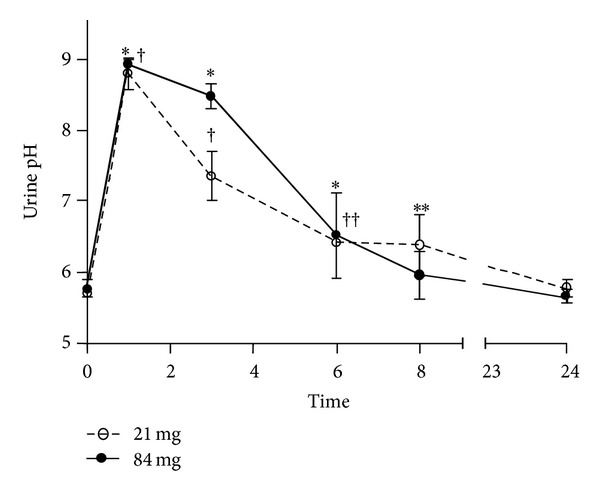
Urine pH in mice after p.o. treatment with sodium bicarbonate. Urine from mice was collected for pH measurements at hours 1, 3, 6, 8, and 24. Urine pH was increased from time 0 to hours 1, 3, 6 (*P < 0.0001), and 8 (**P = 0.05) after treatment with 21 mg of sodium bicarbonate. In mice treated with 84 mg of sodium bicarbonate, urine pH was increased at hours 1, 3 (^†^ *P* < 0.0001), and 6 (^††^ *P* = 0.053).

Previous studies have demonstrated that chronic administration of 200 mM sodium bicarbonate was sufficient to increase interstitial tumor pHe in mice [15, 16] due to elevated bicarbonate ion [HCO_3_^−^] in the extracellular space. The effect of transient sodium bicarbonate dosing on tumor pHe is currently being investigated but is not yet reported. The experiments discussed here were carried out to indirectly measure the passage of systemic [HCO_3_^−^] excess from a single oral dose of sodium bicarbonate. Urine was collected from mice (*n* = 7) prior to bicarbonate dosing. Mice were p.o.-treated with a single dose of 21 mg or 84 mg of sodium bicarbonate. Urine was collected for pH measurements after 1, 3, 6, 8, and 24 hours. The results from the dosing experiments showed that urine pH peaked by the first hour after dosing with both concentrations, raising urine pH from predosing levels (5.8 ± 0.09) by over three pH units (8.9 ± 0.05). Mice treated with 21 mg sodium bicarbonate exhibited an average urine pH of 7.3 ± 0.13 after 3 hours, while average urine pH of mice dosed with 84 mg of sodium bicarbonate was 8.64 ± 0.09. Between 6 and 8 hours, urine pH decreased to near pretreatment levels of 6.6 ± 0.19 in both treatment groups, signaling the approximate endpoint of transient alkalinization in mice. Mice completely cleared excess bicarbonate by hour 24, exhibiting pretreatment urine pH levels (5.7 ± 0.04) (Figure 2). 

### 3.3. Introducing Sodium Bicarbonate Intraperitoneally Affects Fluorescent Signal from Protease Activatable Agents

**Figure 3:**
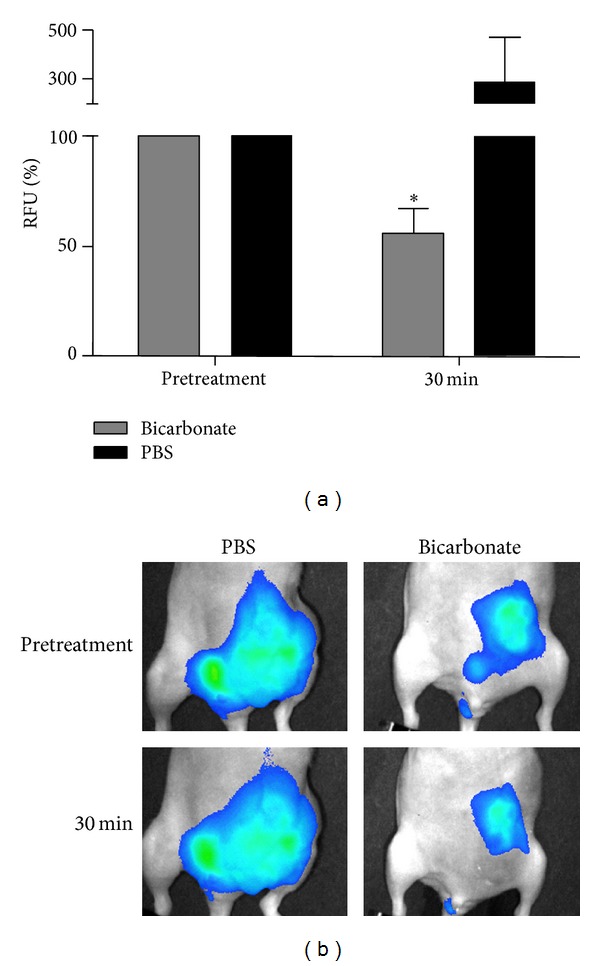
Effect of i.p. sodium bicarbonate administration on cathepsin activity-related fluorescence in mouse xenografts. (a) Cathepsin activity in tumor bearing mice (*n* = 3) expressed as a percentage of the fluorescence taken prior to injecting 1 mL of sodium bicarbonate (1 M) or 1 mL of PBS. The change in fluorescence in PBS-treated mice was not statistically significant (*P* = 0.3). The mean 43.7% reduction in fluorescence of the sodium bicarbonate-treated group was statistically significant (*P = 0.002). (b) Representative images comparing a PBS-treated mouse to a sodium bicarbonate-treated mouse.

These experiments were carried out to determine if i.p. injection of sodium bicarbonate would modulate the fluorescent signal from tumor-specific protease activatable agents. The class of contrast agents used for the experiments is not fluorescent until enzymatic conversion releases a fluorochrome which is detected in the near-infrared region (700–1000 nm). The near-infrared spectrum penetrates tissues more efficiently than visible light or infrared protons. The resulting images demonstrate high-quality target-to-background fluorescence signals [20]. Nude mice were used to establish MDA-MB-231-GFP mammary xenografts to avoid background signal in the GFP spectra caused by hair. Tumor GFP expression was used to correlate tumor signal with fluorescence signal from injectable agents and test for adverse effects of the experimental treatments on fluorescence signals unrelated to the specific markers. Animals injected with protease activatable, pan-cathepsin fluorescent agent were imaged at time 0 and then received a single 1 mL i.p. injection of 1 M sodium bicarbonate (equivalent to 84 mg) or PBS sham treatment. Fluorescence intensities in individual mice were normalized to time 0 (pretreatment = 100% fluorescence intensity). At 30 minutes, the fluorescence signal from bicarbonate-injected mice decreased to 56 ± 6% (*P* = 0.002) (Figures 3(a) and 3(b)). Although the variability between signals was high between PBS-treated mice, there was no significant change in fluorescence between the two imaging times (*P* = 0.3) (Figures 3(a) and 3(b)). 

**Figure 4:**
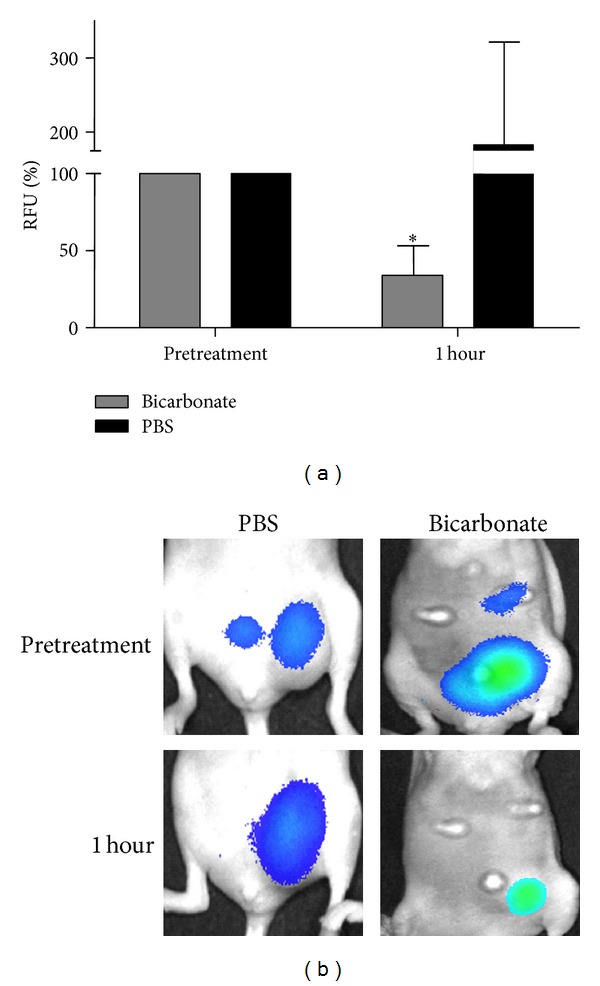
Effect of i.p. sodium bicarbonate administration on MMP activity-related fluorescence in mouse xenografts. MMP activity in tumor bearing mice expressed as a percentage of the fluorescence taken prior to injecting 1 mL of 1 M sodium bicarbonate (*n* = 5) or PBS (*n* = 3). The mean change in fluorescence 1 hour after injection of sodium bicarbonate was statistically significant (*P < 0.0001). (b) Representative images comparing a PBS-treated mouse to a sodium bicarbonate-treated mouse.

Similar experiments were carried out in tumor bearing mice injected with a matrix metalloprotease (MMP) activatable agent. As previously discussed, mice received i.p. administration of 1 M sodium bicarbonate or PBS after a 0 time image acquisition and then imaged 1 hour after treatment. MMP activity-based fluorescence decreased in all mouse tumors to 34 ± 9% after 1 hour in bicarbonate-treated mice (*P* < 0.0001). MMP-related fluorescence was unchanged after 1 hour in PBS-treated mice (Figures 4(a) and 4(b)). The GFP signal in the tumors was unaffected by i.p. injection of sodium bicarbonate (see Supplementary Figure  1 in the Supplementary Material available online at http://dx.doi.org/10.1155/2013/485196).

### 3.4. Introducing Sodium Bicarbonate Orally Affects Fluorescent Signal from Protease Activatable Agents

**Figure 5:**
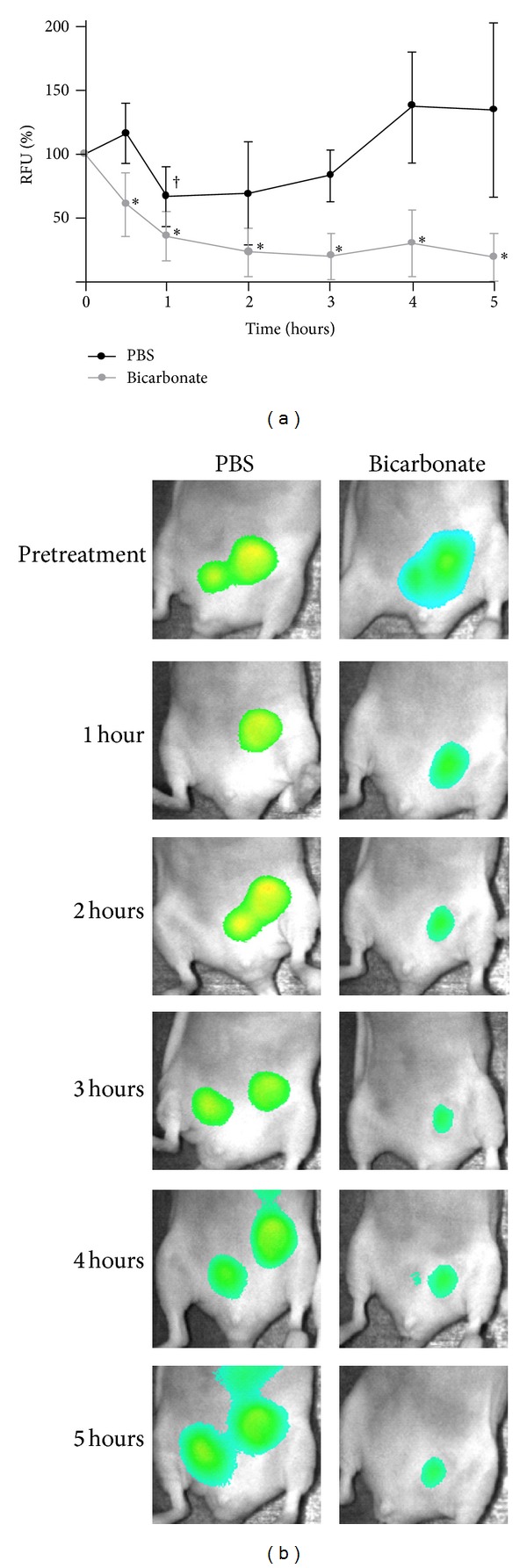
Effect of oral sodium bicarbonate administration on cathepsin activity-related fluorescence in mouse xenografts. Tumor bearing mice (*n* = 6) were imaged prior to treatment and then received 42 mg of sodium bicarbonate or PBS (p.o.). (a) Subsequent images were taken at 30 minutes, 1, 2, 3, 4, and 5 hours. Fluorescence-based activity is expressed as a percentage of signals taken prior to treatment. The reduction in fluorescence-based activity was statistically significant in sodium bicarbonate-treated mice at all time points (*P ≤ 0.003). No significant change in activation-based fluorescence was observed in PBS-treated mice except at the 1-hour time point (^†^ *P* = 0.014). (b) Representative image of experiment comparing PBS-treated mouse to bicarbonate-treated mouse.

These experiments sought to evaluate fluorescent signal responses from protease activatable agents after an oral administration of sodium bicarbonate to tumor bearing mice. After pretreatment imaging, mice were p.o.-treated with 42 or 84 mg of sodium bicarbonate mixed in 0.25 mL water or 0.25 mL PBS. Bicarbonate-treated mice injected with pan-cathepsin fluorescent agent exhibited a significant reduction (*P* ≤ 0.003) in activity-based tumor fluorescence signal to 61 ± 10% in the first 30 minutes decreasing to 36 ± 8% by the first hour. Fluorescence levels remained at about 23 ± 4% between hours 2 and 5 (Figure 5). Although there was high variability in the fluorescence signals generated from PBS-treated mice, fluorescence levels was unchanged after the pretreatment image time point with the exception of the first hour when mean fluorescence decreased to 67 ± 12% (*P* = 0.014) (Figure 5). 

**Figure 6:**
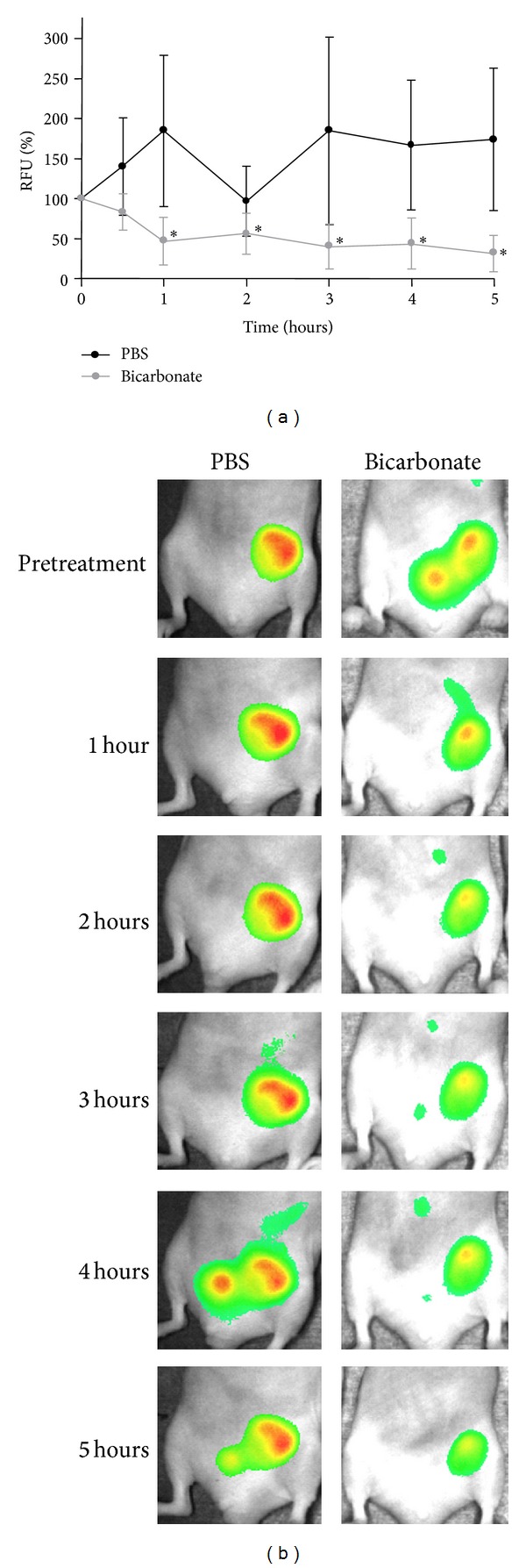
Effect of oral sodium bicarbonate administration on MMP activity-related fluorescence in mouse xenografts. Tumor bearing mice (*n* = 6) were imaged prior to treatment and then received 42 mg of sodium bicarbonate or PBS (p.o.). (a) Subsequent images were taken at 30 minutes, 1, 2, 3, 4, and 5 hours. Fluorescence-based activity is expressed as a percentage of signals taken prior to treatment. The reduction in fluorescence-based activity was statistically significant in sodium bicarbonate-treated mice after the 30-minute time point (*P ≤ 0.003). (b) Representative image of experiment comparing PBS-treated mouse to bicarbonate-treated mouse.

Similar outcomes were observed in bicarbonate-treated mice injected with the MMP activatable agent. Between hours 1 and 5, tumor fluorescence had decreased to 43 ± 5% of the pretreatment time point of each mouse (*P* ≤ 0.003) (Figure 6). As with the i.p. administration experiments, the GFP signal in the tumors of sodium bicarbonate-treated mice was unchanged by the treatments (Supplementary Figure  1).

In separate experiments where mice received 84 mg of sodium bicarbonate, the results showed that this dose was effective in producing a reduction in protease activity-based tumor fluorescence after pretreatment image acquisition. Activity-based fluorescence decreased to 52 ± 7% at the 3-hour time point and to 29 ± 13% by hour 6 in pan-cathepsin-injected mice (*P* ≤ 0.006) (Supplementary Figure  2(a)). Bicarbonate-treated mice injected with MMP activatable agent exhibited a reduction of tumor fluorescence to 12 ± 4% after the first hour and 38 ± 4% by hour 2 (*P* < 0.0003) (Supplementary Figure  2(b)). Activation-based fluorescence in PBS-treated mice was unchanged in both of these experiments (Supplementary Figure  2). 

### 3.5. Sodium Bicarbonate Alkalinization Does Not Affect Carbonic Anhydrase IX Tumor Expression *In Vivo*

**Figure 7:**
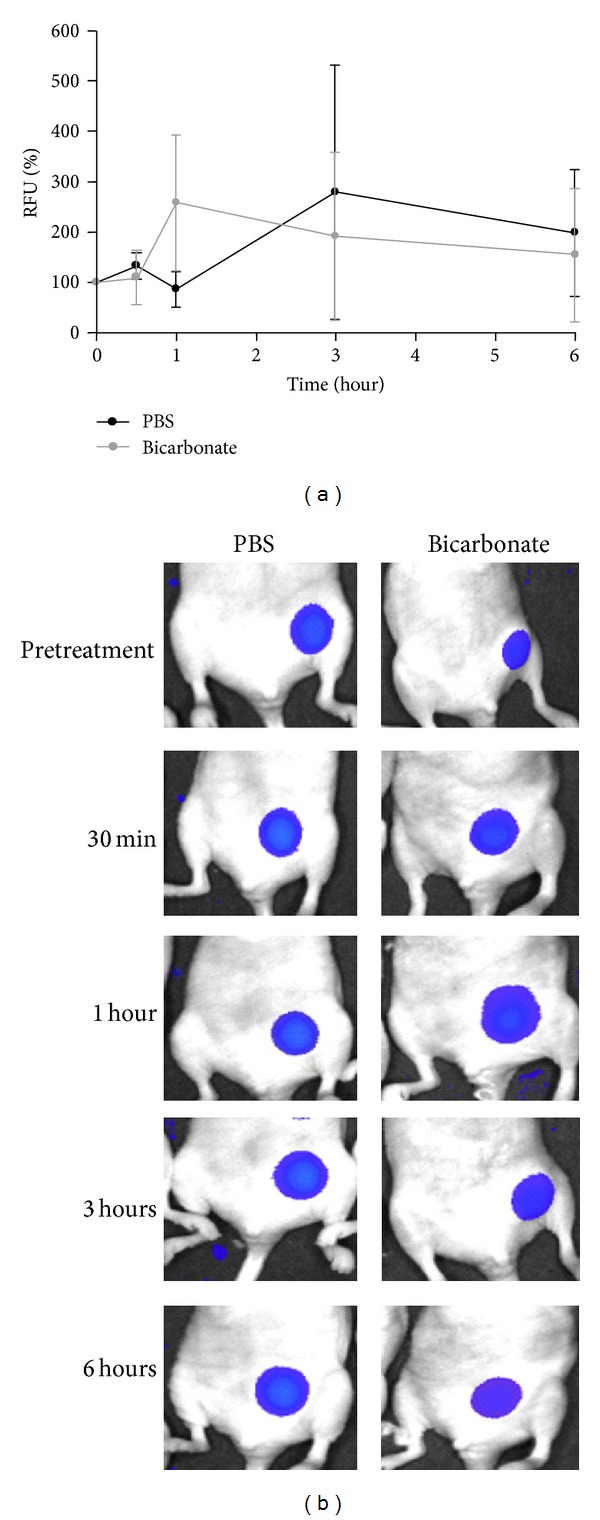
Effect of sodium bicarbonate on CAIX expression-related fluorescence. (a) Tumor bearing mice (*n* = 3) received 84 mg of sodium bicarbonate or PBS p.o. immediately after pretreatment imaging. Subsequent images were acquired at 30 minutes, 1, 3, and 6 hours. CAIX expression is expressed as a percentage of the fluorescence taken prior to treatment. (b) Representative image of experiment comparing PBS-treated mouse to sodium bicarbonate-treated mouse.

These experiments sought to determine if systemic alkalinization through oral sodium bicarbonate administration would regulate the expression of tumor CAIX. Noninvasive imaging was carried out using a CAIX targeted fluorescence agent. Tumor bearing mice were p.o.-treated with 84 mg of sodium bicarbonate mixed in 0.25 mL water or 0.25 mL PBS after the pretreatment imaging course. Expression of CAIX in tumors was unchanged in treated and control mice over a 6-hour imaging period (Figure 7). 

## 4. Discussion

The studies presented here attempt to demonstrate a potential mechanistic association between alkalinization and the subsequent reduction of metastases and improved survival in breast tumor bearing mice. The hypothesis for these studies was that sodium bicarbonate administration produces excess systemic bicarbonate, which in turn alkalinizes the interstitial tumor space. Alkalinization is proposed to inhibit tumor invasion through the modulation of cell surface proteases involved in tumor invasion processes.

Previous studies have considered that systemic alkalinization may inhibit tumor colonization or extravasation into occult sites, but findings from experiments of bicarbonate-treated, tumor bearing mice yielded mixed results. The evidence that alkalinization inhibits the rate of tumor cell extravasation was shown in experiments where metastatic tumor cells (MDA-MB-231) were injected into mouse spleens to measure tumor invasion into the liver [16]. The findings do support the role of alkalinization in inhibiting tumor colonization from one organ to another, but that role is less clear under conditions where the tumor cells are present in the vasculature. For example, bicarbonate-treated mice with PC3M tumors exhibited marginally, albeit significantly, fewer metastases than untreated mice after a 35-day period [16]. Bicarbonate treatment in mice with tail vein-injected B16 tumors, however, did not have a reduced metastatic load compared to the untreated cohort [16].

Earlier investigations evaluating the role of alkalinization in inhibiting intravasation were inconclusive [16]. The reasons are not known, but we note that the flow cytometry experiments measuring CTC blood concentrations were carried out in mice with relatively small MDA-MB-231 mammary xenografts (121 mm^3^). The current studies allowed the tumors to grow to a much larger volume (~1,800 mm^3^), before measuring for CTCs. The rationale for this methodological revision was to maximize the probability of measuring higher numbers of CTCs at least in the untreated cohort. Our findings, which showed a greater than 2-fold higher frequency of CTCs in untreated mice, suggest that sodium bicarbonate treatment may inhibit primary tumor invasion and intravasation into the vasculature. We predict that this effect occurs through the alkalinization of the interstial space of the primary tumor which modulates enzymatic activity of pH-sensitive enzymes involved in regulating tumor invasion processes. 

Tumor expressing MMPs or lysosomal proteases may be inhibited by alkalinization of tumor pHe. Certainly, *in vitro* experiments have confirmed that cathepsin B activity is regulated by extracellular pH [5, 9, 16, 21]. Other studies have shown as well that optimal activity of cysteine proteases resides at a lower pH and may be inactivated under neutral pHe conditions [5, 9, 21, 22]. The role of pH modulation in MMPs is less certain. With the exception MMP-3, which is optimally active at pH 6.0 [23], the activity of most MMPs is optimal under physiological pH (7.4) [24–27]. Mathematical modeling experiments suggest that MMP activity is not mediated by acidic pH and therefore may not be subject to modulation by alkalinization [22]. However, there are numerous reports linking the role of pHe modulation and MMP activity in cancer. Culturing various types of murine and human tumor cell lines in acidic media conditions results in a higher concentration of active form of MMPs [4, 5, 21, 28–30], implicating a pH-sensitive component associated with these enzymes, whereby the effect of *in vivo* pHe buffering or alkalinization could diminish activity levels. It has also been reported that MMP activity may be mediated by cathepsin B in a pH-dependent fashion, supporting an indirect role for alkalinization in reducing MMP activity [21]. 

 We proposed to verify tumor protease activity *in vivo* using fluorescent activatable agents pertaining to cathepsins and MMPs. These agents do not directly measure cathepsin or MMP activity. Fluorescence signals are rather an indirect indicator of upstream protease activity. Due to the short fluorescent life spans of these agents *in vivo*, longitudinal studies in mice chronically treated with sodium bicarbonate in drinking water were not feasible. Therefore, experiments evaluated the activity-related tumor region fluorescence in response to transient alkaline dosing. 

To demonstrate the passage of excess system [HCO_3_^−^], urine pH was monitored in mice dosed with a single bolus of sodium bicarbonate. Transient dosing with sodium bicarbonate increases urine pH over 3 log units within an hour, and this change diminishes moderately over the course of 6–8 hours in mice. While these experiments do not directly measure tumor pHe, they provide some characteristics about the rapid passage and diffusion of systemic [HCO_3_^−^] after an oral dose. Additionally, the urine pH experiments were useful for informing the guidelines for subsequent experiments testing activatable fluorescent agents in response to transient alkalinization. 

Fluorescent activatable agents corresponding to MMPs and cathepsins exhibit a measurable and significant decrease in fluorescent signal as a result of systemic sodium bicarbonate administration. Route of administration does not appear to change the outcome. Fluorescence signal was not significantly changed in the subsequent imaging time points after the pretreatment image acquisition in PBS-treated mice, but there was an instance where fluorescence signal measured significantly lower after 1 hour from PBS treatment. Although this phenomenon cannot be fully explained, it suggests that the precision of data obtained in whole body imaging experiments with these agents may be limited. Future studies should focus on the role of these agents and pH modulation of the tumor microenvironment at higher magnification. 

Since other studies have shown that pH modulation could regulate expression of numerous tumor enzymes [5], we investigated the *in vivo* expression of carbonic anhydrase IX (CAIX) in mice treated with sodium bicarbonate. Membrane CAIX is a major regulator of intracellular pH and is commonly expressed in invasive tumor cells [31–33]. Its activity on the cell surface results in the production of protons [H^+^] into the extracellular milieu [34]. Transient alkalinization did not appear to affect CAIX expression in tumor bearing mice. The findings are similar to other studies investigating tumor CAIX expression after chronic treatment with sodium bicarbonate (200 mM) in which alkalinization does not appear to have an effect on enzyme expression [35]. Alkalinization may be more effective at the earliest stages of tumor development to effectively reduce expression of CAIX [18]. It is unknown if alkalinization directly regulates the activity of CAIX. Overall, the findings suggest that alkalinization may inhibit tumor invasion from the primary site in part by suppressing the activity of invasion enzymes, but does not appear to modulate enzyme expression within a short time frame.

The role of alkalinization in cancer is a controversial subject and not well understood. There is some reporting on the palliative role of alkalinization through sodium bicarbonate treatment in cancer patients [36, 37], but most studies examining alkalinization in cancer treatment focus on animal models for breast and prostate cancer [15–19, 35, 38]. Although diet-induced, endogenous acid production is a relevant factor in chronic diseases [39–41] and may have a potential role in cancer [39, 42], it is not yet clear if dietary alkaline loading helps to prevent cancer or diminishes tumor aggression. 

Acidity appears to be paradoxically stressful, yet an important driver of tumor progression and aggression [43]. Interestingly, chronic alkalinization in spontaneous tumor models prevents cancer onset compared to untreated controls [18] and reduces the expression of tumor autophagic markers that signal tumor survival responses to stress [35]. These data indicate that neutralizing a stressful physiological condition such as acidity mitigates phenotypes associated with aggressive cancer. The overall findings should encourage a wider investigation into the role alkalinization or net-base diets as a beneficial component in prevention, treatment, or maintenance of cancer. 

## 5. Conclusion

In conclusion, the findings presented here offer insight into the mechanisms of alkalinization in inhibiting the spread of tumor metastases. Our observations warrant further, more quantitative investigations on enzymatic activity or expression in response to pH modulation at the level of the tumor microenvironment. Future studies should also evaluate the effect of alkalinization on the invasion of other acidic tumor types, the effect of alkalinization on the activity of other pH-sensitive tumor enzymes, and the efficacy of other types of alkaline agents against tumor acidity.

## Supplementary Material

We considered that administration of sodium bicarbonate might directly affect fluorescence signals in a manner unrelated to enzymatic activity. To account for this possibility, we measured fluorescence from GFP-expressing tumors in response to sodium bicarbonate treatments at the highest dosage of 84 mg per mouse. The GFP signal in the tumors was unaffected by i.p. injection and p.o. administration of sodium bicarbonate in the time ranges that were tested (Supplementary Figure 1).
We also evaluated a dose of 84 mg sodium bicarbonate in a separate set of *in vivo* experiments testing the effect of transient alkalinization on the signals from protease activatable fluorescent agents. We report that mean activity-based fluorescence decreased from 100% at the pretreatment time point to 52 ± 7% at the 3-hour time point and to 29 ± 13% by hour 6 in mice injected with the pan-cathepsin agent (P ≤ 0.006) (supplementary Figure 2(a)). Similarly, mice receiving 84 mg of bicarbonate and injected with MMP activatable agent exhibited reduced mean tumor fluorescence of 12 ± 4% of the pretreatment fluorescent value at hour 1, and 38 ± 4% by hour 2 (P < 0.0003) (supplementary Figure 2(b)). Activation-based fluorescence in PBS treated mice, however, was unchanged in both of these experiments (Supplementary Figure 2).
Supplementary Figure 1. Effect of sodium bicarbonate treatment on tumor GFP signal. Tumor bearing mice (*n* = 3) were imaged before i.p. or p.o. treatments with sodium bicarbonate (84 mg). The changes in GFP fluorescence over time were not statistically significant.
Supplementary Figure 2. Effect of p.o. treatment with sodium bicarbonate on cathepsin and MMP activity-related fluorescence. Tumor bearing mice were imaged prior to treatment then received 84 mg of sodium bicarbonate or PBS (p.o.). Fluorescence-based activity is expressed as a percentage of signals taken prior to treatment. The reduction in fluorescence-based activity was statistically significant in sodium bicarbonate treated mice after A) the pre-treatment time point in ProSense^®^ FAST (pan-cathepsin) treated mice (*n* = 4) (^*∗*^P ≤ 0.006), and B) in MMPSense^*™*^ 750 FAST treated mice (*n* = 3) (^*∗∗*^P ≤ 0.00025). No significant change in activation-based fluorescence was observed in PBS treated mice.
