# Supplementary material for: Investigating Mechanisms of Alkalinization for Reducing Primary Breast Tumor Invasion
Source: Biomed Res Int. 2013 Jul 10;2013:485196. doi: 10.1155/2013/485196 (PMC3722989; doi:10.1155/2013/485196)
Supplement: Supplementary file 1 — We considered that administration of sodium bicarbonate might directly affect fluorescence signals in a manner unrelated to enzymatic activity. To account for this possibility, we measured fluorescence from GFP-expressing tumors in response to sodium bicarbonate treatments at the highest dosage of 84 mg per mouse. The GFP signal in the tumors was unaffected by i.p. injection and p.o. administration of sodium bicarbonate in the time ranges that were tested (Supplementary Figure 1). We also evaluated a dose of 84 mg sodium bicarbonate in a separate set of in vivo experiments testing the effect of transient alkalinization on the signals from protease activatable fluorescent agents. We report that mean activity-based fluorescence decreased from 100% at the pretreatment time point to 52 ± 7% at the 3-hour time point and to 29 ± 13% by hour 6 in mice injected with the pan-cathepsin agent (P ≤ 0.006) (supplementary Figure 2(a)). Similarly, mice receiving 84 mg of bicarbonate and injected with MMP activatable agent exhibited reduced mean tumor fluorescence of 12 ± 4% of the pretreatment fluorescent value at hour 1, and 38 ± 4% by hour 2 (P < 0.0003) (supplementary Figure 2(b)). Activation-based fluorescence in PBS treated mice, however, was unchanged in both of these experiments (Supplementary Figure 2). Supplementary Figure 1. Effect of sodium bicarbonate treatment on tumor GFP signal. Tumor bearing mice (n = 3) were imaged before i.p. or p.o. treatments with sodium bicarbonate (84 mg). The changes in GFP fluorescence over time were not statistically significant. Supplementary Figure 2. Effect of p.o. treatment with sodium bicarbonate on cathepsin and MMP activity-related fluorescence. Tumor bearing mice were imaged prior to treatment then received 84 mg of sodium bicarbonate or PBS (p.o.). Fluorescence-based activity is expressed as a percentage of signals taken prior to treatment. The reduction in fluorescence-based activity was statistically significant in sodium bicarb [file 485196.f1.docx]

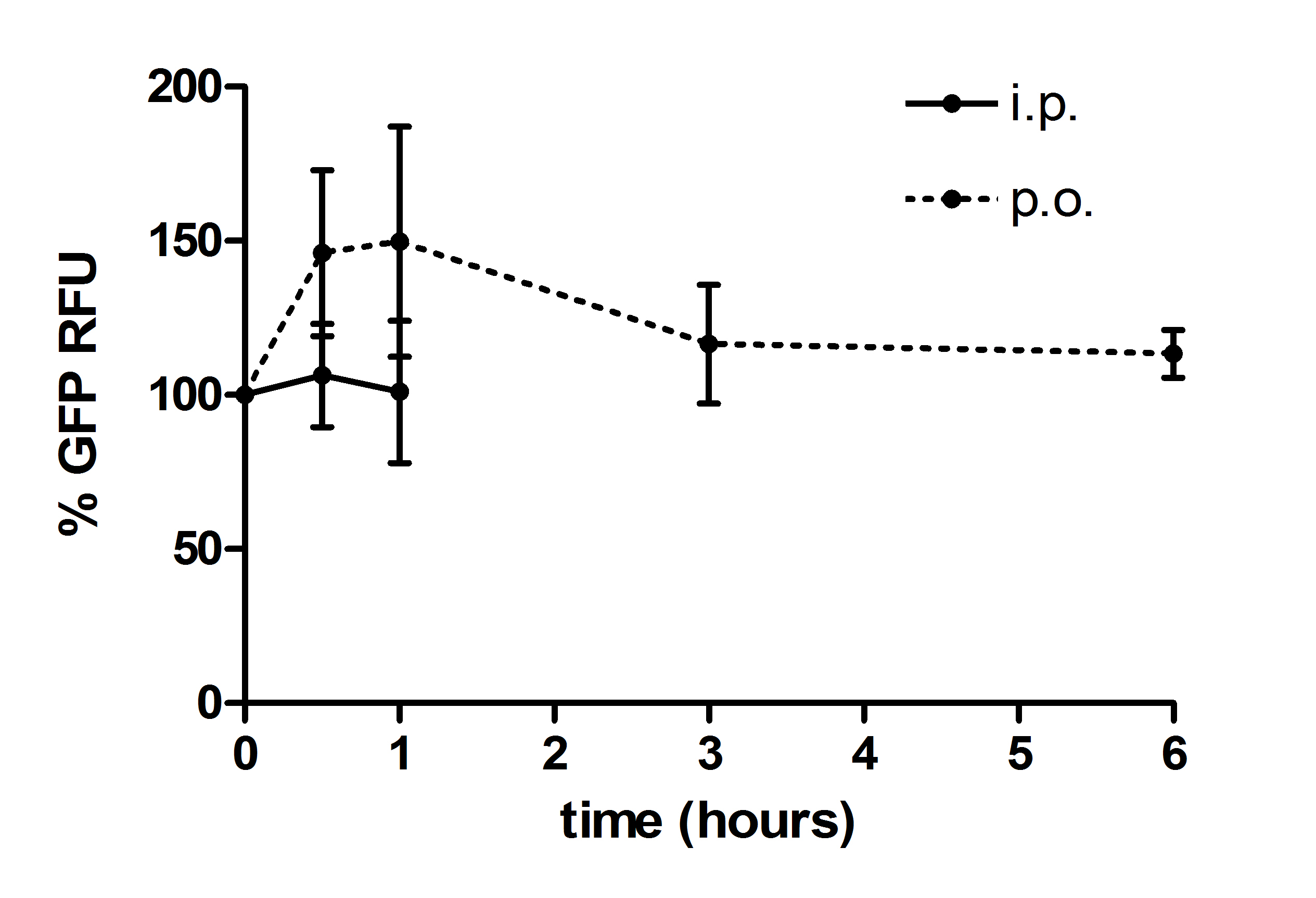


**Supplementary Figure 1. Effect of sodium bicarbonate treatment on tumor GFP signal**. Tumor bearing mice (*n* =3) were imaged before i.p. or p.o. treatments with sodium bicarbonate (84 mg). The changes in GFP fluorescence over time were not statistically significant.
