# Supplementary material for: Investigating Mechanisms of Alkalinization for Reducing Primary Breast Tumor Invasion
Source: Biomed Res Int. 2013 Jul 10;2013:485196. doi: 10.1155/2013/485196 (PMC3722989; doi:10.1155/2013/485196)
Supplement: Supplementary file 2 [file 485196.f2.docx]

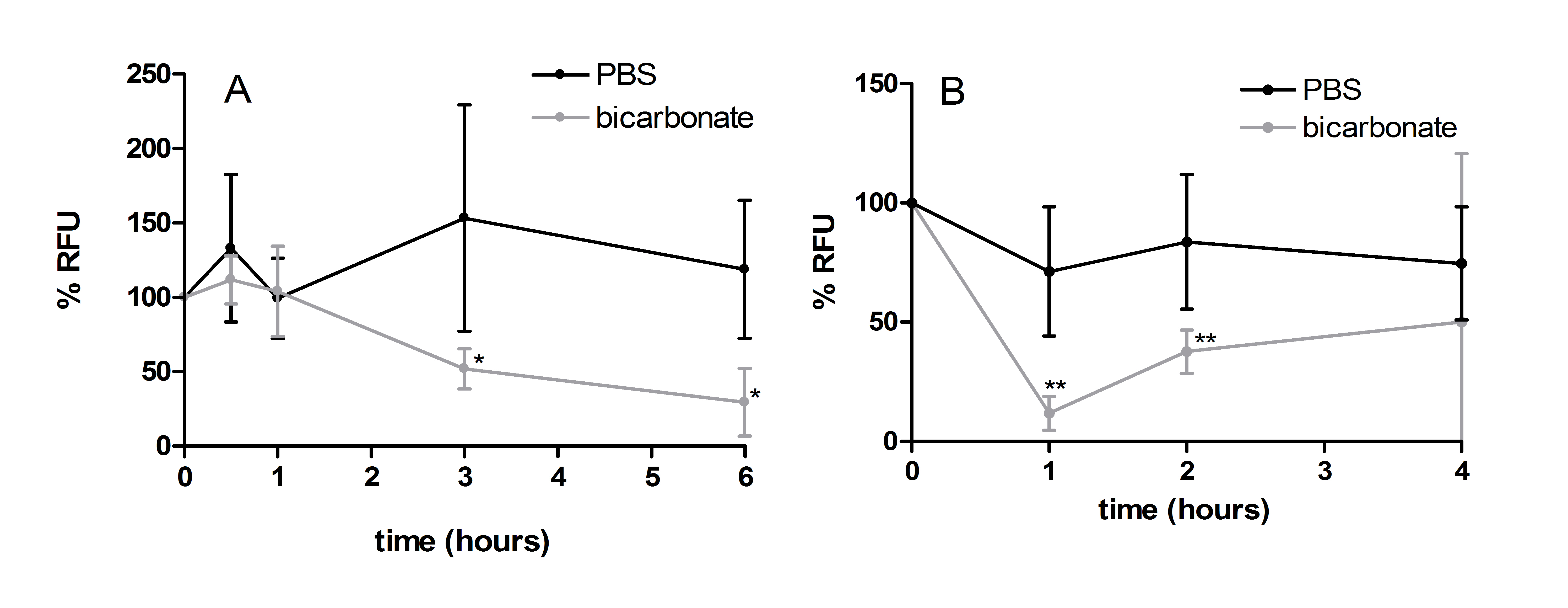


**Supplementary Figure 2. Effect of p.o. treatment with sodium bicarbonate on cathepsin and MMP activity-related fluorescence**. Tumor bearing mice were imaged prior to treatment then received 84 mg of sodium bicarbonate or PBS (p.o.). Fluorescence-based activity is expressed as a percentage of signals taken prior to treatment. The reduction in fluorescence-based activity was statistically significant in sodium bicarbonate treated mice after A) the pre-treatment time point in ProSense® FAST (pan-cathepsin) treated mice (*n* = 4) (**P* ≤ 0.006), and B) in MMPSense™ 750 FAST treated mice (*n* = 3) (***P* ≤ 0.00025). No significant change in activation-based fluorescence was observed in PBS treated mice.
